# Lymphocytic choriomeningitis virus: an unrecognized teratogenic pathogen.

**DOI:** 10.3201/eid0104.950410

**Published:** 1995

**Authors:** L L Barton, C J Peters, T G Ksiazek

**Affiliations:** University of Arizona Health Sciences Center, Department of Pediatrics and Steele Memorial Childrens Research Center, Tuscon, Arizona, USA.


### Lymphocytic Choriomeningitis Virus: An Unrecognized Teratogenic Pathogen

Lymphocytic choriomeningitis virus (LCMV), the first member of the arenavirus family to be isolated, is the causative agent of a zoonosis acquired from chronically viremic mice or hamsters (1). The clinical spectrum of acquired human LCMV infection ranges from inapparent and asymptomatic to, in rare instances, severely symptomatic, systemic, and fatal central nervous system (CNS) disease. Intrauterine LCMV infection has resulted in fetal or neonatal death, as well as hydrocephalus and chorioretinitis in infants (2-6). We have diagnosed congenital LCMV infection in three infants (7) and have collated published and unpublished data on three additional affected infants (8, G.R. Istre, pers. comm.). This report briefly summarizes the salient features of the infection in five of these six American infants and outlines the similarities between these and features observed earlier in Europe. We suggest that LCMV is a more frequent cause of CNS disease in newborns than previously recognized.

Congenital LCMV infection was first recognized in Great Britain in an infant who died at 12 days of age (3). Subsequently, fetal infection with spontaneous abortion (2) and congenital infection in liveborn infants with hydrocephalus and chorioretinitis were documented in Germany (4), France (6), and Lithuania (5). We have recently documented congenital LCMV infection in three infants from Arizona (7) and have obtained information regarding three additional neonates from Arizona, Nebraska (8), and Texas (G.R. Istre, pers. comm.). Detailed clinical and laboratory data are available for five of the six infants. All displayed nonobstructive hydrocephalus with periventricular calcifications, chorioretinitis, and psychomotor retardation. One of the five infants had sensorineural deafness. None of the infants had cardiac abnormalities. Two infants have had follow-up ophthalmologic and audiologic examinations which have shown neither the progression of chorioretinitis nor the development of new auditory deficits. *Toxoplasma gondii*, cytomegalovirus, *Herpes simplex* virus, rubella, enterovirus, and *Treponema pallidum* infections were excluded by culture or serology in all infants. The diagnosis of congenital LCMV infection was confirmed in all infants by immunofluorescence antibody (IFA) and enzyme-linked immunosorbent assays (ELISAs). In addition, serum, CSF, urine, and throat wash specimens from two infants were injected into Vero cell monolayers. Neither cytopathic effect nor LCMV antigens were detected after incubation. Because virus isolation was only attempted after the disease was first diagnosed when the children were 10 months of age, failure to isolate LCMV was not unanticipated.

Laboratory diagnosis of LCMV infection is generally made by serologic techniques. IFA is a more sensitive diagnostic method than either complement fixation or neutralizing antibody techniques (9,10). The newer ELISAs are now being used to evaluate congenitally infected infants. Testing the child's serum and CSF and a simultaneously obtained serum specimen from the mother yields the maximum information if done as soon after birth as possible.

The mothers of four of the five infants in this report had a history of febrile illness during pregnancy, in contrast to a minority of mothers of affected infants previously reported. Typical LCMV infection in adults is a biphasic disease with fever, malaise, myalgias, anorexia, nausea, vomiting, pharyngitis, cough, and adenopathy followed by defervescence and a second phase of CNS disease. However, CNS symptoms may appear without any prodrome or may never develop. Meningitis and meningoencephalitis are the most frequent neurologic manifestations of disease, although myelitis, Guillain-Barré syndrome, and sensorineural deafness have been reported (11). Between 1941 and 1958, 8% to 11% of viral CNS syndromes in hospitalized patients in a Washington, D.C., medical center were etiologically associated with LCMV (12). Arthritis, parotitis, orchitis, myocarditis, and rash have also been noted (13). Clinical interest in LCMV, however, has not been maintained, and the disease is rarely considered despite improved serodiagnostic methods.

Although a history of contact with rodents and their excreta is of diagnostic utility, it is not universally present. A maternal history of rodent exposure was elicited for three of our five infants. Wild mice (*Mus musculus*) and hamsters infected in utero with LCMV during maternal viremia develop both persistent viremia and viruria. The virus is transmitted to humans by direct animal contact; by contact with infected rodent saliva, nasal secretions, urine, feces, semen, and milk; and by infectious aerosols (1). Human-to-human transmission has not been documented. The distribution of LCMV is highly variable within mouse populations. Seasonal, annual, and cyclical variations in rodent density and infection have been postulated but remain inadequately studied (14). LCMV spreads to humans in rural settings or when human habitats are substandard. Infected laboratory and pet rodents have also been associated with disease in humans (1). Serologic surveys and clinical studies have documented both epidemic and endemic human infection in Europe and the Americas. In Baltimore, 9.0% of house mice and 4.7% of residents have had measurable LCMV antibody (15,16).

We hypothesize that congenital LCMV infection is generally undiagnosed and may account for unexplained hydrocephalus with microcephaly or macrocephaly, deafness, blindness, and mental retardation (three of the five infants in this report were referred for infectious disease consultation by pediatric geneticists, and two were referred by pediatric neurologists). No accurate data are available regarding the prevalence and persistence of LCMV antibodies in unselected infants, children, and adults in diverse geographic locales or in children with unexplained visual and/or auditory deficits, microcephaly, and retardation. Increased recreational activities in rural environments, rehabilitation of and habitation in older rodent-infected domiciles, and acquisition of unscreened rodents for pets or laboratory use pose as yet undefined risks for LCMV infection to the fetus, child, and adult. The need for further research to define the frequency of LCMV infection in human and animal populations is clear. LCMV infection can best be prevented by educating the public and medical professionals on the hazards of contact with infected rodents.

**Leslie L. Barton, M.D.,* C.J. Peters, M.D.,†**
**T.G. Ksiazek, D.V.M., Ph.D.†**
*University of Arizona Health Sciences Center, Department of Pediatrics and Steele Memorial Childrens Research Center, Tucson, Arizona, USA;
†National Center for Infectious Diseases, Centers for Disease Control and Prevention, Atlanta, Georgia, USA

### Acknowledgments

We thank Drs. Jane Wilson, Gregory Istre, Stephen Chartrand, and Laurie Seaver for patients' information; Dr. Matafija Seinbergas for his enthusiastic support; and Ms. Amy Sites for technical support.

### References

1. Jahrling PB, Peters CJ. Lymphocytic choriomeningitis: a neglected pathogen of man. Arch Pathol Lab Med 1992;116:486-8.
2. Ackermann R, Stammler A, Armbruster B. Isolierung von Virus der lymphozytaren Choriomeningitis aus Abrasionsmaterial nach Kontakt der Schwangeren mit einem Syrischen Goldhamster (*Mesocricetus auratus*). Infection 1975;3:47-9.
3. Komrower GM, Williams BL, Stones PB. Lymphocytic choriomeningitis in the newborn: probable transplacental infection. Lancet 1955;1:697-8.
4. Ackermann R, Korver G, Turss R, Wonne R, Hochgesand P. Prenatal infection with the lymphocytic choriomeningitis virus. Dtsch Med Wochenschr 1974;13:629-32.
5. Seinbergas MM. Hydrocephalus due to prenatal infection with the lymphocytic choriomeningitis virus. Infection 1976;4:185-91.
6. Chastel C, Bosshard S, Le Goff F, Quillien MC, Gilly R, Aymard M. Infection transplacentaire par le virus de la choriomeningite lymphocytaire: resultats dune enguete serologique retrospective en France. Nouv Press Med 1978;7:1089-92.
7. Barton LL, Budd SC, Morfitt WS, et al. Congenital lymphocytic choriomeningitis virus infection in twins. Pediatr Infect Dis J 1993;12:942-6.
8. Larsen PD, Chartrand SA, Tomashek KY, Hauser LG, Ksiazek TG. Hydrocephalus complicating lymphocytic choriomeningitis virus infection. Pediatr Infect Dis J 1993;12:528-31.
9. Lewis VJ, Walter PD, Thacker WL, Winkler WG. Comparison of three tests for the serological diagnosis of lymphocytic choriomeningitis virus infection. J Clin Microbiol 1975;2:193-7.
10. Lehmann-Grube F, Kallay M, Ibscher B, Schwartz R. Serologic diagnosis of human infections with lymphocytic choriomeningitis virus: comparative evaluation of seven methods. J Med Virol 1979;4:125-36.
11. Lehmann-Grube F. Diseases of the nervous system caused by lymphocytic choriomeningitis virus and other arenaviruses. In: Handbook of clinical neurology. New York: Elsevier, 1989;12:355-81.
12. Meyer HM, Johnson RT, Crawford, IP, Dascomb HE, Rogers NG. Central nervous system syndromes of "viral" etiology: a study of 713 cases. Am J Med 1960:334-47.
13. Lewis JM, Utz JP. Orchitis, parotitis and meningoencephalitis due to lymphocytic-choriomeningitis virus. N Engl J Med 1961;265:776-80.
14. Childs JE, Peters CJ. Ecology and epidemiology of arenaviruses and their hosts. In: The Arenaviridae. New York: Plenum, 1993:331-84.
15. Childs JE, Glass GE, Korch GW, Ksiazek TG, Leduc JW. Lymphocytic choriomeningitis virus infection and house mouse (*Mus musculus*) distribution in urban Baltimore. Am J Trop Med Hyg 1992;47:27-34.
16. Childs JE, Glass GE, Ksiazek TG, Rossi CA, Barrera Oro JG, Leduc JW. Human-rodent contact and infection with lymphocytic choriomeningitis and Seoul viruses in an intercity population. Am J Trop Med Hyg 1991;44:117-21.
